# A hierarchical machine learning model for predicting self-harm and suicidal behaviour in hospitalised patients with schizophrenia using clinical history and nursing observations

**DOI:** 10.3389/fpsyt.2026.1760380

**Published:** 2026-05-15

**Authors:** Chen Liang, Xianfeng Meng, Ying Duan, Wei Yang, Gang Zhu, Jinhuan Wang, Ying Sun, Mingtao Wang, Miao Liu, Chenhui Sun, Kunyuan Hu, Wei Shao, Jintao Ren, Xiaojun Shao, Yang Zhang

**Affiliations:** 1 Key Laboratory of Networked Control Systems, Chinese Academy of Sciences, Shenyang, China; 2 Shenyang Institute of Automation, Chinese Academy of Sciences, Shenyang, China; 3 University of Chinese Academy of Sciences, Beijing, China; 4 Liaoning Provincial Mental Health Center, Tieling, Liaoning, China; 5 Liaoning Maternal and Child Health Hospital, Shenyang, Liaoning, China; 6 Department of Psychiatry, The First Affiliated Hospital of China Medical University, Shenyang, Liaoning, China; 7 Shengjing Hospital of China Medical University, Shenyang, Liaoning, China

**Keywords:** clinical history, hierarchical model, machine learning, nursing observations, risk prediction, schizophrenia, self-harm, suicidal behaviour

## Abstract

**Objective:**

This study aimed to develop and evaluate a two-layered machine learning framework that combines admission clinical information with longitudinal nursing observations to identify schizophrenia inpatients at high risk of self-harm or suicidal acts.

**Methods:**

We retrospectively reviewed the records of 477 patients with schizophrenia hospitalised in Liaoning Province between July 2021 and July 2024. According to whether at least one self-injurious or suicidal episode was documented during the index admission, 159 individuals were assigned to a high-risk group and 318 to a non-high-risk group. At admission, 18 baseline variables (including age, sex, history of self-harm, hopelessness/depression, and educational attainment) were extracted from electronic medical records, and 39 nurse-rated behavioural items were scored weekly using the Psychiatric Patient Nursing Observation Scale. Static and dynamic feature sets were used to train six classifiers [regularized logistic regression (LR), support vector machine (SVM), extreme gradient boosting, random forest, multi-layer perceptron, and K-nearest neighbours]. The best static model (regularized LR) and the best dynamic model (SVM) were combined through probability-level weighted fusion to generate a hierarchical risk score.

**Results:**

Multivariable analysis of admission features showed that previous self-harm [odds ratio (OR) = 4.323], hopelessness/depression (OR = 3.090), younger age (OR = 0.938), and higher educational level (OR = 1.357) were independent predictors of self-harm/suicidal behaviour. Among dynamic indicators, negative self-evaluation (OR = 2.303), self-reported depression (OR = 1.812), insomnia (OR = 1.768), talking to oneself (OR = 1.733), crying (OR = 1.700), and reduced conversation with others (OR = 1.422) remained significant. The optimised static LR model achieved an area under the curve (AUC) of 0.7564, and the dynamic SVM model reached an AUC of 0.8531. Their fusion further improved performance (AUC = 0.9048; sensitivity 0.8542; specificity 0.7789; accuracy 0.8042). This hierarchical model outperformed the best flat combined-feature model (SVM; AUC = 0.9022) in sensitivity (0.8542 vs. 0.6667), indicating a more clinically appropriate detection of high-risk patients.

**Conclusion:**

A hierarchical machine learning approach that integrates baseline clinical history with repeated nursing assessments can effectively flag schizophrenia inpatients at high risk for self-harm and suicidal behaviour, supporting timely and individualised preventive strategies in psychiatric wards.

## 1 Introduction

Schizophrenia is a chronic and disabling psychotic illness in which perception, thinking, emotion, and behaviour are profoundly disturbed. Suicide represents a major cause of premature death in this group and is a key public health concern. Meta-analyses indicate that roughly 5% of patients with schizophrenia eventually die by suicide, and between one quarter and one half attempt suicide at least once during their lifetime (1, 2). Recent population-based studies have shown that, compared with the general population, individuals with schizophrenia have several-fold higher suicide mortality, with the highest absolute and relative risks typically seen in younger adults (3). Within psychiatric hospitals, suicidality is particularly prominent: pooled estimates suggest that about one third of inpatients with schizophrenia report current suicidal ideation, and a substantial proportion of suicides occur either during admission or shortly after discharge (4, 5). Together, these findings highlight the need for practical and accurate tools to identify inpatients with schizophrenia who are at elevated risk of self-harm or suicidal acts.

Research over the past decades has delineated a broad range of correlates of suicidal thoughts and behaviours in schizophrenia. *Static* risk factors that reflect relatively enduring vulnerabilities include younger age, previous suicide attempts or episodes of self-harm, depressive or affective symptomatology, repeated psychiatric hospitalisations, and co-occurring substance use disorders (2, 6). Whereas these risk markers tend to remain stable over the course of illness, *dynamic* or state-dependent variables are more closely tied to short-term changes in suicidal risk. Examples of the latter include the onset or worsening of hopelessness and depressive symptoms, agitation, insomnia, withdrawal from social contact, and intensification of psychotic experiences (1, 5). In everyday clinical work, however, these static and dynamic sources of information are often considered separately and in a subjective way. Clinicians may informally combine historical risk markers with current symptoms, but systematically integrating many features from both domains in busy ward settings remains difficult.

To assist clinicians, several structured instruments have been designed to assess suicide risk. Widely used examples include the Beck Scale for Suicide Ideation (BSS/BSSI) and the Columbia-Suicide Severity Rating Scale (C-SSRS) (7, 8). These tools help to standardise the collection of information about suicidal ideation and behaviour, but systematic reviews indicate that their predictive performance is only modest and that they have limited ability to distinguish which patients will later engage in self-harm or suicide (7, 9). More recently, actuarial models such as the Oxford Mental Illness and Suicide (OxMIS) tool have been developed to estimate suicide risk in people with severe mental disorders using a prespecified set of clinical and sociodemographic variables (10, 11). Nevertheless, such approaches mainly rely on static baseline information and provide only a single snapshot of risk; they do not take advantage of the rich longitudinal behavioural data routinely available during inpatient treatment, for example repeated nursing observations of mood, social interaction and daily functioning.

Machine learning (ML) methods offer a complementary route to suicide risk prediction and can, in principle, exploit large and heterogeneous clinical datasets more effectively than traditional scales. A rapidly expanding literature has applied ML techniques to electronic health records, narrative clinical notes and other routinely collected information to forecast suicidal ideation, suicide attempts and suicide deaths (12, 13). Studies based on large general or psychiatric cohorts have reported that ML models can outperform clinician judgement and conventional risk scores in distinguishing high-risk individuals, particularly when structured clinical variables are combined with behavioural or text-derived features (14–16). However, the field remains actively debated: systematic reviews have noted that many published models suffer from high or unclear risk of bias, limited external validation, and predictive accuracy that does not consistently surpass traditional risk scales (17, 18), and the clinical utility of such models has rarely been demonstrated (12). While many of these models have been developed in broad psychiatric or general-population cohorts—a valid approach for population-level risk stratification— relatively few have focussed specifically on people with schizophrenia or on the inpatient setting where risk is especially acute (13). A notable exception is the work of Qiao et al. (19), who applied ML models to nurse-rated behavioural data from 131 schizophrenia inpatients and found that structured nursing observations contributed meaningfully to suicide risk prediction, although the small sample limited generalisability. In addition, most published ML models adopt what might be termed a *flat* architecture, in which static and dynamic predictors are entered together as a single feature vector rather than being modelled in separate stages. Although such models can, in principle, capture interactions between enduring vulnerabilities and short-term symptom changes through learned feature combinations, they do not make this distinction explicit, which may limit clinical interpretability and the ability to trace how baseline risk is modified by recent behavioural fluctuations.

In our previous work on violence risk, we tackled a related problem by proposing a hierarchical ML model that combines static clinical history with dynamic nursing observations to predict violent incidents in hospitalised patients with schizophrenia (20). In that framework, long-term vulnerability (static risk) and short-term behavioural change (dynamic risk) were captured by two separate sub-models, and their outputs were merged using a decision-level weighted fusion strategy. This design improved both predictive performance and interpretability compared with conventional flat models and mirrored the way clinicians tend to think about risk: first forming an impression based on background history, then modifying it according to recent changes in the patient’s clinical state. Although self-harm/suicidal behaviour and interpersonal violence are distinct outcomes with different clinical implications, both can be understood as arising from the interplay between persistent vulnerability factors and acute state-dependent triggers.

The present study builds directly on this prior work by adapting the hierarchical modelling framework from interpersonal violence to self-harm and suicidal behaviour in schizophrenia inpatients. Using a larger inpatient cohort, we combine admission-time static variables—including history of self-harm, hopelessness or depression, age and educational level—with dynamic indicators derived from weekly nurse-rated observation scales, such as negative self-evaluation, self-reported depression, crying, insomnia, talking to oneself and reduced interaction with others. By reusing a previously validated architecture while changing the outcome and refining the feature sets, we seek to: (i) examine whether the same structural separation of static and dynamic risk information can yield accurate predictions of self-harm and suicidal behaviour; (ii) compare the independent risk factors and their relative contributions with those identified for violent behaviour; and (iii) develop a clinically interpretable, data-driven tool that leverages routine nursing observations to support timely, personalised preventive interventions in psychiatric inpatient care.

The main contributions of this paper can be summarised as follows. First, we assemble a detailed inpatient dataset that links electronic medical records with high-resolution nursing observation data to characterise self-harm and suicidal behaviour among hospitalised patients with schizophrenia. Second, we extend a previously validated hierarchical ML framework to this new outcome, explicitly separating and recombining static and dynamic risk components at the decision level. Third, we show that the resulting model attains strong discriminative performance for self-harm and suicidal behaviour and yields an interpretable profile of long-term vulnerabilities and short-term warning signs that can inform targeted clinical risk management.

## 2 Materials and methods

This section provides an overview of the study design, data sources, and modelling workflow used to build a hierarchical predictor of self-harm and suicidal behaviour in inpatients with schizophrenia. We begin by describing the clinical setting and sampling strategy, specifying the inclusion and exclusion criteria and summarising the baseline characteristics of the study cohort. We then outline how the data were collected and organised, distinguishing admission-time clinical information (static features) from behavioural information repeatedly rated by nurses during hospitalisation (dynamic features). The definition of the outcome is introduced next, together with the procedure used to assign patients to the high-risk or non-high-risk group according to documented episodes of self-injury or suicide attempts.

Subsequently, we summarise the main preprocessing operations undertaken before model building, including data cleaning, handling of missing values, coding of categorical variables, feature scaling, and splitting the dataset into separate training and test sets. The following subsections then detail the specification of the hierarchical model: construction of baseline statistical and machine-learning models for static and dynamic features, hyperparameter tuning based on cross-validation, and combination of model outputs at the decision level. Finally, we describe the performance metrics and analytical procedures used to evaluate the models on the held-out test set and to examine their generalisability and potential usefulness for clinical risk assessment.

### 2.1 Study population

#### 2.1.1 Study setting and eligibility criteria

We carried out a retrospective, observational study in a psychiatric hospital in Liaoning Province that provides specialised care for patients with schizophrenia. Using the hospital’s electronic medical record (EMR) system, we identified all admissions to the schizophrenia wards between July 2021 and July 2024. Individuals were eligible for inclusion if they satisfied the following criteria: (1) a principal diagnosis of schizophrenia recorded according to the International Classification of Diseases, 10th Revision (ICD–10); (2) age 18–65 years at admission; (3) an inpatient stay exceeding 2 weeks; and (4) completion of at least two weekly evaluations with the Psychiatric Patient Nursing Observation Scale during the index admission.

We excluded patients with serious or unstable physical disease, organic brain pathology, or other neurological disorders that could markedly influence the evaluation of psychiatric symptoms or self-harm/suicidal behaviour. To ensure that the dynamic behavioural features represented the period prior to any self-harm event, we also discarded cases in which the first recorded self-injurious or suicidal act occurred during the initial week of hospitalisation, as these patients lacked sufficient pre-event nursing observation data for model development. After applying these inclusion and exclusion rules, 477 inpatients with schizophrenia formed the final analytic sample for subsequent analyses.

#### 2.1.2 Demographic and clinical baseline characteristics

A total of 477 inpatients with schizophrenia were included in the final analyses. Their mean age was 41.62 ± 10.43 years, and by design all participants were between 18 and 65 years old. Of the whole cohort, 260 patients (54.51%) were male and 217 (45.49%) were female. Most patients were unmarried (75.26%), and the majority were recorded as being employed at admission (82.39%). Regarding educational level, 229 individuals (48.01%) had completed high school or higher education, whereas 248 (51.99%) had finished junior high school or less. The median duration of illness was 8 years, with an interquartile range of 4–18 years. The mean length of the index hospitalisation was 4.46 ± 1.87 weeks. A history of substance abuse was present in 156 patients (32.70%), and 151 patients (31.66%) had previously attempted suicide or engaged in self-harm.

### 2.2 Data collection

#### 2.2.1 Data sources and composition

The study relied entirely on information routinely recorded in the hospital’s clinical information systems. Two main data streams were used: (i) the electronic medical record (EMR) system of the hospital and (ii) nurse-rated scores from the Psychiatric Patient Nursing Observation Scale. These sources together yielded two complementary feature sets. From the EMR, we obtained static variables, including sociodemographic characteristics and baseline clinical history documented at admission. From the nursing observation scale, we derived dynamic variables based on weekly ratings throughout the inpatient stay, reflecting short-term fluctuations in symptoms, daily functioning, and behaviour on the ward. The subsequent subsections describe the specific variables included in each feature set and the rationale for their selection.

#### 2.2.2 Static features: clinical history

Eighteen admission-time variables were extracted retrospectively from the EMR to represent each patient’s baseline clinical profile. These *static* features covered sociodemographic information and longstanding clinical history, and comprised: age, sex, marital status, educational level, employment status, personality traits (e.g. introverted vs. extroverted), duration of illness, history of alcohol or drug misuse, previous suicide attempts or episodes of self-harm, the presence of high-risk command hallucinations, persecutory delusions, disordered thought processes, abnormalities of sensation and perception, clinician ratings of overall intellectual functioning, attentional problems, memory impairment, and the presence of hopeless or depressed mood and manic symptoms at admission.

The choice of these 18 variables was informed by prior meta-analytic and clinical evidence linking demographic factors (younger age, social disadvantage), clinical history (previous self-harm, substance misuse, hopelessness), psychotic symptoms (command hallucinations, persecutory delusions), and cognitive indicators to suicide risk in schizophrenia and severe mental illness (1, 2, 6, 11, 21).

#### 2.2.3 Dynamic features: nursing observation scale

Dynamic behavioural information was obtained using the *Psychiatric Patient Nursing Observation Scale*, which is a structured rating form routinely completed by ward nurses. The instrument is derived from the classic Nurses’ Observation Scale for Inpatient Evaluation (NOSIE–30), originally created by Honigfeld and colleagues to quantify behaviour in psychiatric inpatients and subsequently validated in multiple clinical and cultural contexts (22–24). Consistent with our previous study on violence risk in schizophrenia (20), we employed an extended version of the scale that expands the original 30 items to 39 by adding clinically salient indicators (e.g. insight into illness, expressed wish for discharge), thereby providing a more detailed picture of day-to-day ward functioning.

For each patient, the primary nurse rates all 39 items once per week on a 4-point scale (0 = normal/not present, 3 = severe/most abnormal), summarising both how often and how intensely the behaviour occurred during the preceding week. Ratings are usually completed on Friday afternoons and are based on continuous observation from Saturday through Friday. The items span a broad range of domains: adherence to ward rules; care of personal belongings; participation in ward activities such as bed-making, cleaning, occupational therapy, recreational programmes and exercise; appropriateness of dress relative to ambient temperature; self-reported physical discomfort; patterns of interaction with family members, peers and staff; emotional expression and responses to humour; personal hygiene (e.g. face washing, tooth brushing, foot washing, hair grooming, toileting and menstrual hygiene); eating habits; and observable psychopathology.

Several item clusters are particularly relevant to self-harm and suicidal risk, including depressive features (low mood, crying, negative self-evaluation), sleep disturbance, social engagement indicators, and psychotic or psychomotor phenomena. Prior work has linked these domains to suicidal ideation and behaviour in schizophrenia (1, 2, 25, 26).

### 2.3 Patient grouping

#### 2.3.1 Operational definition of self-harm and suicidal behaviour

In this study, the outcome of interest was self-directed self-harm that occurred during the index hospitalisation. We adopted a broad definition of self-harm consistent with WHO and NICE guidance (27, 28): any intentional act of self-poisoning or self-injury during the index hospitalisation, irrespective of stated suicidal intent. An event was coded as present when (1) the act was directed against the patient’s own body with clear potential to cause physical harm, (2) clinicians judged the act to be deliberate, and (3) the episode was documented in the medical record as self-harm, a suicide attempt, or deliberate self-injury.

Included behaviours: Examples of acts classified as self-harm/suicidal behaviour included, but were not limited to: intentional self-cutting or stabbing of the skin; attempted hanging, strangulation, or suffocation using ligatures or other materials; jumping or attempting to jump from a height or into traffic; deliberate overdose of prescribed or over-the-counter medications beyond therapeutic dosage; intentional ingestion of toxic substances (such as disinfectants, pesticides, or other chemicals); and other clearly documented deliberate acts aimed at injuring oneself. Episodes were coded as present regardless of whether they resulted in medical treatment, loss of consciousness, or serious physical complications, provided that the potential for harm was evident.

Excluded behaviours: To maintain specificity, several types of behaviour were not classified as self-harm/suicidal behaviour. These included purely verbal expressions of suicidal ideation (e.g., statements about wanting to die) without any accompanying self-injurious act; indirect or passive self-endangering behaviours such as refusal of food or medication in the absence of an acute self-harm act; and repetitive, stereotyped self-injury associated with severe neurodevelopmental disorders (e.g., habitual mild head banging) when clearly documented as such in the record and not judged by clinicians as deliberate self-harm. Accidental injuries and unintentional overdoses were also excluded.

#### 2.3.2 Grouping process and criteria

A structured, multi-step procedure was used to assign patients to the *high-risk* (self-harm/suicide) or *non-high-risk* groups based on the above operational definition.

1. Raters and calibration: Two attending psychiatrists with experience in suicide risk assessment served as independent raters. Prior to the chart review, they jointly reviewed the study’s operational definition of self-harm/suicidal behaviour and discussed example vignettes to ensure a shared understanding of inclusion and exclusion criteria, drawing on established terminology from self-directed violence classification systems and suicide risk assessment tools (27, 29, 30).
2. Record review and event identification: For each participant, the raters independently examined the complete electronic medical record for the index hospitalisation, including admission notes, progress notes, nursing shift reports, and adverse event or incident forms. The review focussed on narrative descriptions of self-directed injury or poisoning. To minimise missed cases, raters also used keyword searches (for example, “self-harm”, “self-injury”, “suicide attempt”, “overdose”, “cut”, “hang”) within the electronic record system to flag potential events for closer inspection.
3. Event coding and agreement: Each identified episode was evaluated against the operational definition in Section 2.3.1 and coded as either meeting or not meeting criteria for self-harm/suicidal behaviour. After both raters had completed their independent reviews, coding sheets were compared. Cases with discrepant ratings were re-examined in a consensus meeting, during which the raters jointly reviewed the original documentation to resolve disagreements. When consensus could not be reached on the presence of a qualifying event, the more conservative judgement (i.e., “no self-harm event”) was adopted.
4. Group assignment: After discrepancies had been resolved, each patient received a final outcome label. Any individual who experienced at least one (≥ 1) episode that satisfied the criteria for self-harm/suicidal behaviour during the index admission was classified as belonging to the high-risk group; patients without any qualifying self-harm or suicidal events were placed in the non-high-risk group. Using this scheme, 159 of the 477 patients were allocated to the high-risk group and the remaining 318 to the non-high-risk group. Baseline demographic and clinical characteristics of these two groups are summarised in **Table 1**.

**Table 1:** Distribution of patient characteristics in self-harm/suicide and non-self-harm/suicide groups.

Characteristics	Category or unit	High-risk group (n=159)	Non-high-risk group (n=318)	Total (n=477)
Age	Years	38.02 ± 8.81	43.42 ± 10.72	41.62 ± 10.43
Duration of illness	Years	7 (4, 16)	9 (4, 18)	8 (4, 18)
Gender	Male	74 (46.54%)	186 (58.49%)	260 (54.51%)
	Female	85 (53.46%)	132 (41.51%)	217 (45.49%)
Marital Status	Married	35 (22.01%)	83 (26.10%)	118 (24.74%)
	Unmarried	124 (77.99%)	235 (73.90%)	359 (75.26%)
Occupation	Employed	129 (81.13%)	264 (83.02%)	393 (82.39%)
	Unemployed	30 (18.87%)	54 (16.98%)	84 (17.61%)
Educational Level	High School or above	90 (56.60%)	139 (43.71%)	229 (48.01%)
	Junior High or below	69 (43.40%)	179 (56.29%)	248 (51.99%)
Substance Abuse History	Yes	62 (38.99%)	94 (29.56%)	156 (32.70%)
	No	97 (61.01%)	224 (70.44%)	321 (67.30%)
Suicide History	Yes	82 (51.57%)	69 (21.70%)	151 (31.66%)
	No	77 (48.43%)	249 (78.30%)	326 (68.34%)

### 2.4 Data preprocessing

#### 2.4.1 Dataset partitioning

For all analyses we worked with a single train–test division of the dataset (N = 477). Using the unique patient identifier, patients were randomly assigned via stratified sampling to a training set (n = 334; 111 high-risk, 223 non-high-risk) and an independent test set (n = 143; 48 high-risk, 95 non-high-risk), preserving the class proportions across both subsets. The training data were used for every stage of model construction—feature screening, univariable and multivariable analyses, model fitting, and hyperparameter optimisation. The test data were held out and not examined until the modelling and fusion procedures had been finalised, at which point they were used once to obtain an unbiased estimate of predictive performance on unseen patients. Keeping this fixed partition throughout all experiments helps to prevent information leakage and makes the reported results easier to reproduce.

#### 2.4.2 Data cleaning

Before constructing the analytical dataset, we performed a series of quality checks to ensure that the data were suitable for analysis. Cases that did not meet the inclusion criteria (e.g., insufficient length of stay, fewer than two completed nursing observation assessments, or missing key diagnostic information) were removed at the screening stage. For the remaining records, free-text entries were standardised where necessary, such as converting narrative descriptions of illness duration into numeric year values. All variables were examined for implausible values (e.g., age outside the 18–65 range, negative duration of illness, or nursing observation scores outside the valid 0–3 scale) and obvious data entry errors; any such values were corrected against the original medical record where possible or treated as missing and imputed as described below. The proportion of missing values across all variables was very low (*<*1% for any individual variable). Nevertheless, to ensure a complete analytic dataset, we used simple, distribution-based imputation: continuous variables (for example, age and duration of illness) were imputed with the median of the training set, and categorical variables (such as marital status, employment, substance abuse history, and suicide history) were imputed with the most frequent category in the training set. This approach preserved the overall structure of the data while limiting the impact of missingness on model fitting.

#### 2.4.3 Data encoding

Before modelling, non-numeric variables were converted into formats suitable for standard machine-learning algorithms. Clinical and sociodemographic variables with two categories (such as sex, marital status, employment status, history of substance misuse, history of self-harm or suicide, and key psychotic symptoms including command hallucinations and persecutory delusions) were represented by binary indicators, with 0 indicating absence and 1 indicating presence. Ordered categorical variables were recoded using integer values that preserved their ranking. For educational attainment, we defined a four-level scale in which 0 denoted primary school or below, 1 denoted junior high school, 2 denoted high school or vocational training, and 3 denoted college or higher, so that larger codes reflected higher education. Personality (introverted vs. extroverted) was also treated as a dichotomous variable and encoded accordingly. All coding schemes were checked manually, and any implausible or out-of-range entries were corrected or removed. The 39 items from the nursing observation scale were already recorded on a 0–3 ordinal scale and therefore did not require additional recoding apart from ensuring that they were stored as numeric variables.

#### 2.4.4 Normalisation

Following encoding, we rescaled continuous and quasi-continuous variables to place them on comparable numerical ranges. This step is particularly important for algorithms that are sensitive to feature scale, such as distance-based methods and gradient-based optimisation procedures. We applied standard z-score normalisation using statistics calculated from the training set only. Specifically, for each continuous feature (including age, illness duration, and the aggregated scores derived from the nursing observation scale), we subtracted the training-set mean and divided by the training-set standard deviation. The resulting standardised features have mean 0 and standard deviation 1 in the training data. The same transformation parameters were then applied to the test set, ensuring that information from the test data did not leak into the training process.

### 2.5 Model construction

#### 2.5.1 Prediction task definition

The aim of the modelling was to predict, for each admission, whether a hospitalised patient with schizophrenia would engage in at least one act of self-harm or suicidal behaviour during the current stay. We treated this as a binary classification problem. The outcome variable was coded as 1 for patients with at least one documented self-harm/suicidal episode (high-risk group) and 0 for those with no such events (non-high-risk group).

To reflect the way clinicians typically combine long-standing background information with short-term clinical changes, we decomposed the prediction into two complementary components and then merged their outputs:

- Static risk model. This component uses the 18 admission-time variables described in Section 2.2.2 as input. It returns a probability *R*_0_ that a given patient belongs to the high-risk group and is intended to capture relatively stable, long-term vulnerability.
- Dynamic behavioural model. This component is based on the 39 behavioural indicators described in Section 2.2.3. For patients who later engaged in self-harm/suicidal behaviour, all weekly ratings recorded *before* the first qualifying event were averaged to form a single 39-dimensional summary vector; for patients without such events, weekly scores across the whole admission were averaged. Given this summary vector, the model outputs a probability *R*_dyn_ that the patient is in the high-risk group, representing the patient’s overall behavioural and symptomatic state during the observation window.

The two risk estimates are then combined at the decision level through a linear fusion:


$$R^{*}=\alpha R_{0}+\left(1-\alpha \right)R_{\text{dyn}},$$

where α∈[0,1] is a weighting parameter chosen on the training data. A probability threshold (default 0.5) applied to R^*^ yields the final binary prediction $\hat{y}$ for each patient ( $\hat{y}=1\text{ if }R^{*}\geq 0.5,\;\hat{y}=0$ otherwise).

Conceptually, this decomposition parallels clinical reasoning: long-term risk factors such as prior suicidal behaviour, chronic depressive symptoms and adverse social circumstances provide a baseline level of concern, which is then modified by current information on mood, sleep, interpersonal functioning and ward behaviour over recent weeks. Explicitly separating “static” and “dynamic” components makes the hierarchical model more transparent and helps clinicians interpret how different sources of information contribute to the final risk estimate.

#### 2.5.2 Statistical association analysis and baseline logistic regression

To obtain a transparent benchmark for self-harm/suicide risk and to identify independent predictors, we first built logistic regression (LR) models. LR is a widely used supervised learning approach for binary outcomes; it represents the logit of the event probability as a linear combination of the predictors and then applies the inverse-logit (sigmoid) function to map this value to the range [0,1] (31). Because model coefficients can be interpreted directly as odds ratios, LR offers an intuitive reference against which more flexible machine-learning methods can be compared (32).

##### 2.5.2.1 Stage 1: univariable screening

We began by performing univariable analyses with two objectives: first, to identify and quantify the individual associations between candidate risk factors and self-harm/suicidal behaviour, providing clinically interpretable effect sizes (odds ratios with 95% confidence intervals) that are informative in their own right; and second, to select a parsimonious set of candidate variables for subsequent multivariable modelling. All statistical analyses in this stage were conducted in Python 3.10 using the pandas, NumPy, SciPy and statsmodels libraries.

1. Univariable group comparisons. For continuous variables, the Kolmogorov–Smirnov test was used to check normality. If the data met normality and homogeneity-of-variance assumptions, differences between the high-risk and non-high-risk groups were examined with independent-samples *t*-tests; when these assumptions were violated, we used the Wilcoxon rank-sum test instead. Categorical variables were compared using chi-square tests or Fisher’s exact test, as appropriate.
2. Univariable logistic regression. To quantify the association between each individual predictor and the outcome, a separate LR model was fitted on the training set for every static and dynamic feature. From these models we derived odds ratios (ORs) and 95% confidence intervals (CIs). Features with *P<* 0.05 in the univariable analyses were regarded as potentially relevant and carried forward to the multivariable stage.

##### 2.5.2.2 Stage 2: multivariable logistic regression

Because univariable associations can be distorted by correlations among predictors, we next constructed multivariable LR models to identify variables that were independently related to self-harm/suicidal risk. Using the Logit function in statsmodels, we fitted separate multivariable models on the training data for static and dynamic feature sets. The dependent variable was the binary self-harm/suicide label (1 = high-risk, 0 = non-high-risk), and the covariates were those features that had reached significance in the univariable screening. Regression coefficients were exponentiated to yield adjusted ORs with 95% CIs; variables with *P<* 0.05 were considered statistically significant. This step produced a compact subset of static and dynamic predictors that contributed independently to the outcome.

##### 2.5.2.3 Stage 3: regularised LR submodels and hierarchical scores

Having identified the independently significant predictors in Stages 1 and 2, we now transition from statistical association analysis to predictive modelling. The regularised LR submodels described below, together with the additional machine-learning classifiers presented in Section 2.5.3, collectively form the predictive component of the hierarchical framework. The predictors retained from Stage 2 were then used to build regularised LR models for static and dynamic risk. These models were implemented with scikit-learn’s LogisticRegression class (31). The static LR submodel received the reduced set of baseline variables and generated a probability score *R*_0_, whereas the dynamic LR submodel used the selected behavioural indicators to produce a dynamic score *R*_dyn_.

Model hyperparameters were tuned on the training set by five-fold cross-validation combined with grid search (33). The grid covered the following settings:

- Penalty (penalty): {11,12,elasticnet};
- Regularisation strength (C): values on a logarithmic scale from 10^−4^ to 10^4^;
- Solver (solver): liblinear, lbfgs, saga;
- Maximum iterations (max_iter): 100 and 200.

For each combination of hyperparameters, we calculated the mean area under the receiver operating characteristic curve (AUC) and accuracy across the five folds and selected the configuration with the highest average performance. The final regularised LR models were then refitted on the full training set using these optimal hyperparameters to obtain stable estimates of *R*_0_ and *R*_dyn_. These LR-based risk scores were used both as interpretable standalone models and as one pair of inputs for the hierarchical fusion strategy described below.

#### 2.5.3 Machine learning model construction

Section 2.5.2 (Stage 3) described the construction of regularised LR submodels for static and dynamic risk. Here we describe five additional classifiers trained on the same prediction task to serve as comparators and to explore whether non-linear algorithms could better exploit potential non-linearities and interactions among predictors: multi-layer perceptron (MLP), random forest (RF), k-nearest neighbours (KNN), support vector machine (SVM), and extreme gradient boosting (XGBoost). Unlike the regularised LR submodels, which used only the variables identified as independently significant in Stages 1 and 2 to maintain parsimony and interpretability, these five classifiers received all 18 static or all 39 dynamic features as input, allowing them to discover complex patterns without prior variable restriction. All models were fitted on the training data only; hyperparameters were tuned by cross-validation, and the final configurations were evaluated on the independent test set.

##### 2.5.3.1 Step 1: static models

For the static feature set, each algorithm was optimised separately:

- MLP: hidden layers and neurons, activation function (ReLU or tanh), L2 penalty, and learning rate.
- Random forest: number of trees (n_estimators), maximum depth (max_depth), candidate features per split (max_features), minimum samples to split (min_samples_split), and minimum samples per leaf (min_samples_leaf).
- KNN: number of neighbours *k* ∈ [3,20], weighting scheme (uniform or distance-based), and distance metric (Euclidean or Manhattan).
- SVM: RBF kernel with soft-margin parameter *C* and kernel width *γ*.
- XGBoost: tree depth, learning rate, number of boosting rounds, row and column subsampling rates, minimum child weight, and regularisation parameters (gamma, reg_alpha, reg_lambda).

For each classifier, the hyperparameter setting with the highest five-fold cross-validated AUC on the training set was chosen, after which the model was refitted on the full training data and its performance assessed on the test set.

##### 2.5.3.2 Step 2: dynamic models

The same training and tuning framework was applied to the dynamic feature matrix, now using the 39-item behavioural vectors as inputs. Each of the five algorithms (MLP, RF, KNN, SVM, XGBoost) was optimised via cross-validation on the training subset and then evaluated on the held-out test subset. Because these dynamic predictors describe week-to-week variation in mood, behaviour and psychotic symptoms, methods capable of modelling complex non-linear structure were expected to be particularly informative in this setting.

##### 2.5.3.3 Step 3: fusion of static and dynamic models

To derive hierarchical predictors, we combined a selected static model and a selected dynamic model at the probability level. Let *R*_0_ denote the probability output from a chosen static classifier (for example, regularised LR) and *R*_dyn_ the corresponding probability from a chosen dynamic classifier (for example, SVM). We then formed a fused score.


$$R^{*}=\alpha R_{0}+\left(1-\alpha \right)R_{\text{dyn}},$$

where *α* ∈ [0,1] controls the relative contribution of baseline versus dynamic information. On the training set, *α* was swept from 0 to 1 in increments of 0.05; for each value we computed the mean AUC using five-fold cross-validation and selected the value that maximised this quantity. The resulting R^*^ provides a combined estimate of self-harm/suicide risk, and applying a default threshold of 0.5 to R^*^ on the test data yielded the final binary prediction $\hat{y}$ for each patient ( $\hat{y}=1\text{ if }R^{*}\geq 0.5,\;\hat{y}=0$ otherwise). This conventional threshold was chosen to provide a uniform decision rule across all models compared in this study (static, dynamic, hierarchical and flat), thereby ensuring that differences in sensitivity and specificity reflect genuine model characteristics rather than threshold selection. Because the primary discrimination metric (AUC) is threshold-independent, the choice of 0.5 does not affect the overall assessment of model performance.

##### 2.5.3.4 Step 4: flat combined-feature baseline

To provide a direct baseline against which the hierarchical fusion strategy can be evaluated, we also trained all six classifiers on the concatenated static and dynamic feature sets. For each patient, the 18 admission-time variables and the 39 aggregated nursing-observation scores were combined into a single 57-dimensional input vector; models were then fitted and tuned on the training data using the same cross-validation procedure described above and evaluated on the independent test set. This flat architecture represents the conventional approach of pooling all available predictors into a single model without distinguishing their temporal origin, and its performance is compared with that of the hierarchical model in Section 3.3.4.

### 2.6 Evaluation metrics

We evaluated model performance using sensitivity (recall), specificity, positive predictive value (PPV), negative predictive value (NPV), overall accuracy, and the area under the receiver operating characteristic curve (AUC), and inspected the confusion matrix. In the context of self-harm/suicide screening, sensitivity is of particular clinical importance because false negatives correspond to missed opportunities for intervention (34). AUC was used as the primary measure of overall discrimination because it is threshold-independent and relatively robust to variations in outcome prevalence (13). PPV and NPV were reported to contextualise prediction performance in light of the low base rate of self-harm events (35). To quantify uncertainty, 95% confidence intervals for all metrics were obtained by bootstrap resampling of the test set (1,000 iterations). To assess calibration, we plotted calibration curves for the key models and summarised weak calibration using the calibration intercept and slope. We additionally reported the Brier score as an overall performance measure and the expected calibration error (ECE) as a bin-based summary of miscalibration (36).

## 3 Results

In this section, we present the main empirical findings of the study on predicting self-harm and suicidal behaviour among inpatients with schizophrenia. We begin by summarising the univariable analyses of static and dynamic features used to screen candidate risk factors. Next, we report the multivariable logistic regression models that isolate independent predictors within each feature set. Finally, we detail the predictive performance of the baseline regularised logistic regression models and the hierarchical machine-learning models on the held-out test sample.

### 3.1 Univariate analysis

#### 3.1.1 Single-factor analysis of static features

Baseline static covariates were obtained from the electronic medical records at the time of admission. We compared these variables between patients who exhibited at least one episode of self-harm/suicidal behaviour during hospitalisation (high-risk group) and those who did not (non-high-risk group). Continuous measures, such as age and duration of illness, were analysed with the Mann–Whitney U test, whereas categorical and ordinal variables were examined using chi-square tests or Fisher’s exact test, as appropriate.

As shown in **Table 2**, several admission characteristics differed significantly between the two groups (*P<* 0.05). High-risk patients were, on average, younger than non-high-risk patients (*P<* 0.001), and a history of self-harm or suicide attempts was markedly more common in the high-risk group (*P<* 0.001). Hopelessness or depressive mood at admission was also more prevalent among high-risk patients (*P<* 0.001). Educational level was associated with self-harm/suicidal behaviour, with a greater proportion of individuals having completed high school or above in the high-risk group than in the non-high-risk group (*P* = 0.010). In addition, gender (*P* = 0.030), the presence of persecutory delusions (*P* = 0.036), and personality traits (*P* = 0.042) showed significant group differences, suggesting that these factors may contribute to the likelihood of self-harm or suicidal acts during the inpatient stay.

**Table 2:** Single-factor analysis of static features between self-harm/suicide and non-self-harm/suicide groups.

Feature	Feature type	Test method	P	Significant
Suicide History	Binary variable	Chi-square test	*<*0.001	Yes
Age	Continuous variable	Mann-Whitney U test	*<*0.001	Yes
Hopelessness/Depression	Binary variable	Chi-square test	*<*0.001	Yes
Education Level	Ordinal categorical variable	Mann-Whitney U test	0.010	Yes
Gender	Binary variable	Chi-square test	0.030	Yes
Persecutory Delusions	Binary variable	Chi-square test	0.036	Yes
Personality	Binary variable	Chi-square test	0.042	Yes

#### 3.1.2 Single-factor analysis of dynamic features

Dynamic behavioural indicators were obtained from weekly nurse-rated Psychiatric Patient Nursing Observation Scale scores. Each of the 39 items was coded on an ordinal scale from 0 to 3. The Mann–Whitney U test or chi-square test was used to examine group differences in these dynamic variables.

Thirteen of the 39 dynamic items showed statistically significant differences between the high-risk and non-high-risk groups (*P<* 0.05), as detailed in **Table 3**. High-risk patients had markedly higher scores for negative self-evaluation and self-reported depression (both *P<* 0.001), indicating a more negative view of themselves and more severe subjective depressive symptoms. Sleep disturbance (insomnia) was also more prominent in the high-risk group (*P* = 0.001). Emotionally expressive behaviours such as crying and talking to oneself occurred more frequently among high-risk patients (*P* = 0.003). Furthermore, differences were observed in interpersonal and social functioning: high-risk patients tended to have less conversation with others (*P* = 0.003), less positive attitudes towards others (*P* = 0.003), and lower interest in their surroundings (*P* = 0.026). Impairments in daily functioning and self-care were also evident, with significantly worse scores for personal affairs management, cooperation with staff, participation in work therapy, foot washing, and neat appearance (all *P* ≤ 0.023). These findings indicate that dynamic changes in mood, social engagement, and daily living activities are closely linked to the occurrence of self-harm/suicidal behaviour.

**Table 3:** Single-factor analysis of significant dynamic features between self-harm/suicide and non-self-harm/suicide groups.

Feature	Feature type	Test method	P	Significant
Negative Self-Evaluation	Ordinal Variable	Mann-Whitney U test	*<*0.001	Yes
Self-reported Depression	Ordinal Variable	Mann-Whitney U test	*<*0.001	Yes
Insomnia	Ordinal Variable	Mann-Whitney U test	0.001	Yes
Conversation with Others	Ordinal Variable	Mann-Whitney U test	0.003	Yes
Crying	Ordinal Variable	Mann-Whitney U test	0.003	Yes
Talking to Self	Ordinal Variable	Mann-Whitney U test	0.003	Yes
Attitude Towards Others	Ordinal Variable	Mann-Whitney U test	0.003	Yes
Personal Affairs Management	Ordinal Variable	Mann-Whitney U test	0.004	Yes
Cooperation with Staff	Ordinal Variable	Mann-Whitney U test	0.005	Yes
Work Therapy Participation	Ordinal Variable	Mann-Whitney U test	0.006	Yes
Foot Washing	Ordinal Variable	Mann-Whitney U test	0.012	Yes
Neat Appearance	Ordinal Variable	Mann-Whitney U test	0.023	Yes
Interest in Surroundings	Ordinal Variable	Mann-Whitney U test	0.026	Yes

### 3.2 Multivariate logistic regression analysis

Based on the univariate screening, we next performed multivariable logistic regression analyses to identify static and dynamic variables that independently predicted self-harm/suicidal behaviour.

#### 3.2.1 Multivariable analysis of static features

Static variables with *P<* 0.05 in the univariate comparisons were entered into a multivariable logistic regression model. As shown in **Table 4**, four baseline characteristics remained independently associated with self-harm/suicidal behaviour after adjustment.

**Table 4:** Multivariate logistic regression of static features predicting self-harm/suicide behaviour.

Feature	Coefficient	P	OR	95% CI
Suicide History	1.464	*<*0.001	4.323	2.508–7.451
Age	-0.064	*<*0.001	0.938	0.912–0.964
Hopelessness/Depression	1.128	*<*0.001	3.090	1.817–5.256
Education Level	0.305	0.026	1.357	1.038–1.774

A history of self-harm or suicide attempts was the strongest predictor (OR = 4.323, 95% CI: 2.508–7.451, *P<* 0.001), indicating that patients with prior self-harm had more than fourfold higher odds of engaging in self-harm/suicidal behaviour during hospitalisation. Younger age was also associated with increased risk (OR = 0.938 per year, 95% CI: 0.912–0.964, *P<* 0.001), suggesting that older patients were less likely to exhibit such behaviour. Admission hopelessness/depression significantly elevated risk (OR = 3.090, 95% CI: 1.817–5.256, *P<* 0.001). Finally, higher educational level was associated with higher odds of self-harm/suicidal behaviour (OR = 1.357 per category increase, 95% CI: 1.038–1.774, *P* = 0.026). Together, these results highlight previous self-harm, younger age, depressive/hopeless mood, and higher education as key baseline risk markers.

#### 3.2.2 Multivariable analysis of dynamic features

Dynamic behavioural variables that demonstrated significant univariate associations were entered into a separate multivariable logistic regression model. As presented in **Table 5**, six dynamic indicators remained independently associated with self-harm/suicidal behaviour.

**Table 5:** Multivariate logistic regression of dynamic features predicting self-harm/suicide behaviour.

Feature	Coefficient	p-value	OR	95% CI
Negative Self-Evaluation	0.834	*<*0.001	2.303	1.679–3.159
Self-reported Depression	0.595	*<*0.001	1.812	1.316–2.495
Insomnia	0.570	*<*0.001	1.768	1.290–2.422
Talking to Self	0.550	0.001	1.733	1.244–2.413
Crying	0.530	0.001	1.700	1.228–2.352
Conversation with Others	0.352	0.022	1.422	1.052–1.921

Higher scores for negative self-evaluation (OR = 2.303, 95% CI: 1.679–3.159, *P<* 0.001), self-reported depression (OR = 1.812, 95% CI: 1.316–2.495, *P<* 0.001), insomnia (OR = 1.768, 95% CI: 1.290–2.422, *P<* 0.001), talking to self (OR = 1.733, 95% CI: 1.244–2.413, *P* = 0.001), and crying (OR = 1.700, 95% CI: 1.228–2.352, *P* = 0.001) were each associated with increased risk of self-harm/suicidal behaviour. In addition, poorer conversation with others (higher score on the “conversation with others” item) was independently related to elevated risk (OR = 1.422, 95% CI: 1.052–1.921, *P* = 0.022). These findings underline the importance of dynamic affective and interpersonal changes—particularly intensifying depressive symptoms, negative self-view, disturbed sleep, and social withdrawal—as short-term warning signs of self-harm/suicidal behaviour.

### 3.3 Performance of hierarchical machine learning models

#### 3.3.1 Performance of static baseline models

To evaluate the predictive value of baseline clinical history alone, we trained six different classifiers on the 18 static features: regularized logistic regression (LR), support vector machine (SVM), random forest (RF), multi-layer perceptron (MLP), *k*-nearest neighbours (KNN), and XGBoost. All models were tuned by cross-validation on the training set and then assessed on the independent test set. The detailed performance metrics are summarised in **Table 6**.

**Table 6:** Predictive performance of static baseline models for self-harm/suicide behaviour.

Model type	AUC	Sensitivity	Specificity	Positive predictive value	Negative predictive value	Accuracy
MLP	0.6107 (0.5107–0.7064)	0.3750 (0.2285–0.5122)	0.7579 (0.6701–0.8427)	0.4390 (0.2857–0.5897)	0.7059 (0.6224–0.7900)	0.6294 (0.5524–0.7063)
RF	0.7406 (0.6547–0.8126)	0.2708 (0.1525–0.4043)	0.8947 (0.8269–0.9490)	0.5652 (0.3571–0.7647)	0.7083 (0.6303–0.7881)	0.6853 (0.6084–0.7552)
KNN	0.6432 (0.5461–0.7303)	0.2500 (0.1333–0.3847)	0.8211 (0.7327–0.8901)	0.4138 (0.2400–0.6000)	0.6842 (0.6000–0.7647)	0.6294 (0.5524–0.7063)
SVM	0.7259 (0.6413–0.8050)	0.3542 (0.2195–0.5000)	0.8737 (0.8064–0.9348)	0.5862 (0.4074–0.7601)	0.7281 (0.6460–0.8070)	0.6993 (0.6224–0.7692)
LR	0.7564 (0.6764–0.8315)	0.5833 (0.4399–0.7144)	0.8316 (0.7548–0.9043)	0.6364 (0.4883–0.7826)	0.7980 (0.7188–0.8723)	0.7483 (0.6783–0.8182)
XGBoost	0.7156 (0.6186–0.8003)	0.5000 (0.3617–0.6364)	0.8105 (0.7253–0.8830)	0.5714 (0.4091–0.7143)	0.7624 (0.6789–0.8367)	0.7063 (0.6294–0.7764)

Chance-level performance on the test set: AUC = 0.500; accuracy = 0.664 (majority-class prediction).

The regularised LR classifier achieved the highest AUC (0.7564), followed by SVM (0.7259) and XGBoost (0.7156); RF, KNN and MLP achieved AUCs of 0.7406, 0.6432 and 0.6107, respectively. These results indicate that a parsimonious LR model based on a few key static predictors is sufficient to capture much of the baseline risk signal and provides a strong static reference model.

#### 3.3.2 Performance of dynamic behavioural models

We then evaluated models trained on the 39 dynamic nursing observation features. Again, six algorithms (LR, SVM, RF, MLP, KNN, XGBoost) were compared, with performance metrics reported in **Table 7**.

**Table 7:** Predictive performance of dynamic behavioural models for self-harm/suicide behaviour.

Model type	AUC	Sensitivity	Specificity	Positive predictive value	Negative predictive value	Accuracy
MLP	0.8241 (0.7463–0.8896)	0.7083 (0.5652–0.8334)	0.7895 (0.7052–0.8646)	0.6296 (0.5094–0.7500)	0.8427 (0.7558–0.9126)	0.7622 (0.6923–0.8252)
RF	0.7961 (0.7145–0.8605)	0.1875 (0.0789–0.3036)	0.9474 (0.8947–0.9891)	0.6429 (0.3636–0.8892)	0.6977 (0.6183–0.7752)	0.6923 (0.6154–0.7692)
KNN	0.6717 (0.5854–0.7573)	0.2917 (0.1750–0.4222)	0.9158 (0.8523–0.9688)	0.6364 (0.4286–0.8236)	0.7190 (0.6393–0.8000)	0.7063 (0.6364–0.7832)
SVM	0.8531 (0.7892–0.9029)	0.4167 (0.2800–0.5610)	0.9474 (0.8979–0.9891)	0.8000 (0.6427–0.9474)	0.7627 (0.6789–0.8390)	0.7692 (0.6923–0.8392)
LR	0.8281 (0.7620–0.8856)	0.5833 (0.4444–0.7273)	0.8105 (0.7292–0.8817)	0.6087 (0.4651–0.7381)	0.7938 (0.7083–0.8710)	0.7343 (0.6643–0.8042)
XGBoost	0.8112 (0.7293–0.8799)	0.5417 (0.4081–0.6905)	0.8947 (0.8295–0.9529)	0.7222 (0.5833–0.8684)	0.7944 (0.7168–0.8738)	0.7762 (0.7063–0.8462)

Chance-level performance on the test set: AUC = 0.500; accuracy = 0.664 (majority-class prediction).

The best-performing dynamic model was the SVM classifier, which achieved an AUC of 0.8531, sensitivity of 0.4167, specificity of 0.9474, PPV of 0.8000, NPV of 0.7627, and accuracy of 0.7692. The LR dynamic model also performed well, with an AUC of 0.8281 and a more balanced trade-off between sensitivity (0.5833) and specificity (0.8105). MLP and XGBoost produced AUCs in the range of 0.81–0.82, whereas RF and KNN showed somewhat lower sensitivity. Overall, these findings demonstrate that dynamic behavioural information carries substantial predictive signal for self-harm/suicidal behaviour, and that SVM and LR models are particularly effective in exploiting this information.

#### 3.3.3 Performance of the integrated hierarchical model

Finally, we integrated the static and dynamic risk estimates using the hierarchical fusion strategy described in Section 2.5.1. We selected the best-performing static classifier (regularised LR, based on AUC) and the best-performing dynamic classifier (SVM), and combined them through probability-level weighted fusion to form the final hierarchical model (LR + SVM). The optimal fusion weight *α* was determined by grid search on the training set, and the fused model was evaluated on the independent test set.

**Figure 1:**
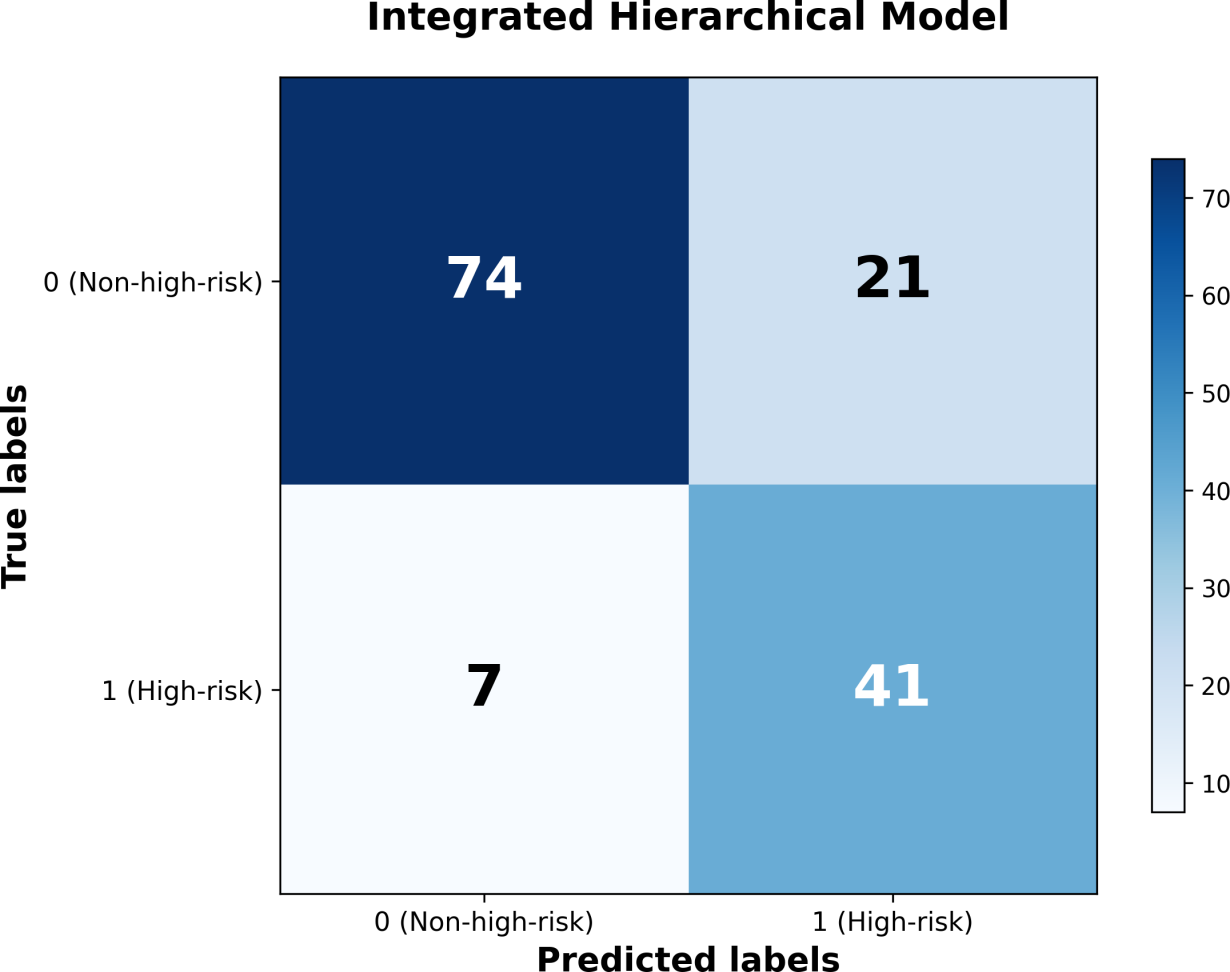
Confusion matrix of integrated hierarchical model predictions.

As shown in **Table 8**, the LR + SVM hierarchical model attained an AUC of 0.9048 (95% CI: 0.8518–0.9472), with sensitivity of 0.8542 (0.7419–0.9435), specificity of 0.7789 (0.6956–0.8603), PPV of 0.6613 (0.5507–0.7797), NPV of 0.9136 (0.8441–0.9697), and accuracy of 0.8042 (0.7413–0.8671) on the test set. The confusion matrix for this model is depicted in **Figure 1**. Among the test-set patients, 41 high-risk individuals were correctly identified (true positives), while 7 high-risk patients were misclassified as low risk (false negatives). Seventy-four non-high-risk patients were correctly classified (true negatives), and 21 non-high-risk patients were incorrectly labelled as high risk (false positives).

**Table 8:** Predictive performance of the integrated hierarchical model for self-harm/suicide behaviour.

Model type	AUC	Sensitivity	Specificity	Positive predictive value	Negative predictive value	Accuracy
LR + SVM	0.9048 (0.8518–0.9472)	0.8542 (0.7419–0.9435)	0.7789 (0.6956–0.8603)	0.6613 (0.5507–0.7797)	0.9136 (0.8441–0.9697)	0.8042 (0.7413–0.8671)

Chance-level performance on the test set: AUC = 0.500; accuracy = 0.664 (majority-class prediction).

These results show that the hierarchical integration of static and dynamic information substantially improves discrimination compared with using either source alone, while maintaining a clinically favourable balance between sensitivity and specificity. The high NPV (0.9136) indicates that patients classified as low risk by the model are very unlikely to engage in self-harm/suicidal behaviour during hospitalisation, whereas the relatively high sensitivity (0.8542) suggests that most high-risk patients can be successfully identified in advance.

#### 3.3.4 Comparison with flat combined-feature models

To evaluate whether the hierarchical fusion strategy offers advantages over simply pooling all predictors, we trained the same six classifiers on the concatenated 57-dimensional feature vector (18 static + 39 dynamic features). Performance metrics for all flat models are summarised in **Table 9**.

**Table 9:** Predictive performance of flat models (57-dimensional concatenated features) for self-harm/suicide behaviour.

Model type	AUC	Sensitivity	Specificity	Positive predictive value	Negative predictive value	Accuracy
MLP	0.8680 (0.8027–0.9226)	0.7083 (0.5714–0.8334)	0.8632 (0.7912–0.9271)	0.7234 (0.5952–0.8432)	0.8542 (0.7812–0.9238)	0.8112 (0.7413–0.8741)
RF	0.8629 (0.7917–0.9185)	0.3750 (0.2391–0.5239)	0.9684 (0.9314–1.0000)	0.8571 (0.6956–1.0000)	0.7541 (0.6774–0.8279)	0.7692 (0.6993–0.8392)
KNN	0.7787 (0.6978–0.8541)	0.4167 (0.2750–0.5601)	0.8737 (0.8061–0.9355)	0.6250 (0.4516–0.7858)	0.7477 (0.6610–0.8261)	0.7203 (0.6434–0.7902)
SVM	0.9022 (0.8471–0.9474)	0.6667 (0.5238–0.8049)	0.9474 (0.8980–0.9892)	0.8649 (0.7561–0.9706)	0.8491 (0.7800–0.9131)	0.8531 (0.7972–0.9091)
LR	0.8680 (0.7941–0.9281)	0.7708 (0.6428–0.8913)	0.8316 (0.7500–0.8977)	0.6981 (0.5714–0.8113)	0.8778 (0.8077–0.9460)	0.8112 (0.7483–0.8671)
XGBoost	0.8107 (0.7234–0.8835)	0.4792 (0.3333–0.6272)	0.8947 (0.8252–0.9529)	0.6970 (0.5312–0.8519)	0.7727 (0.6937–0.8447)	0.7552 (0.6853–0.8252)

Chance-level performance on the test set, AUC = 0.500; accuracy = 0.664 (majority-class prediction).

Among the flat models, the SVM classifier achieved the highest AUC (0.9022), followed by MLP and LR (both 0.8680). Notably, the best flat model (SVM) attained an AUC comparable to that of the hierarchical LR_static_+SVM_dynamic_ model (0.9048 vs. 0.9022). However, the two approaches differed markedly in their sensitivity–specificity trade-off at the default 0.5 decision threshold. The flat SVM achieved high specificity (0.9474) but substantially lower sensitivity (0.6667), meaning that approximately one-third of high-risk patients were missed. In contrast, the hierarchical model achieved a sensitivity of 0.8542 while maintaining adequate specificity (0.7789), resulting in a more clinically favourable balance for a screening context in which false negatives—missed self-harm or suicidal acts—carry greater consequence than false positives. The hierarchical model also yielded a higher NPV (0.9136 vs. 0.8491), indicating that patients classified as low risk were more reliably free of self-harm/suicidal behaviour.

These results suggest that, while flat models can achieve comparable overall discrimination, the hierarchical fusion strategy produces a decision boundary that is better suited to clinical self-harm/suicide screening. In addition, the hierarchical architecture provides an interpretability advantage that flat models cannot offer: by outputting separate probability estimates for static (*R*_0_) and dynamic (*R*_dyn_) risk, clinicians can distinguish whether a patient’s elevated risk originates primarily from enduring vulnerability factors or from recent behavioural deterioration—information that is clinically actionable but unavailable from a single combined-feature score.

#### 3.3.5 Calibration analysis

**Figure 2:**
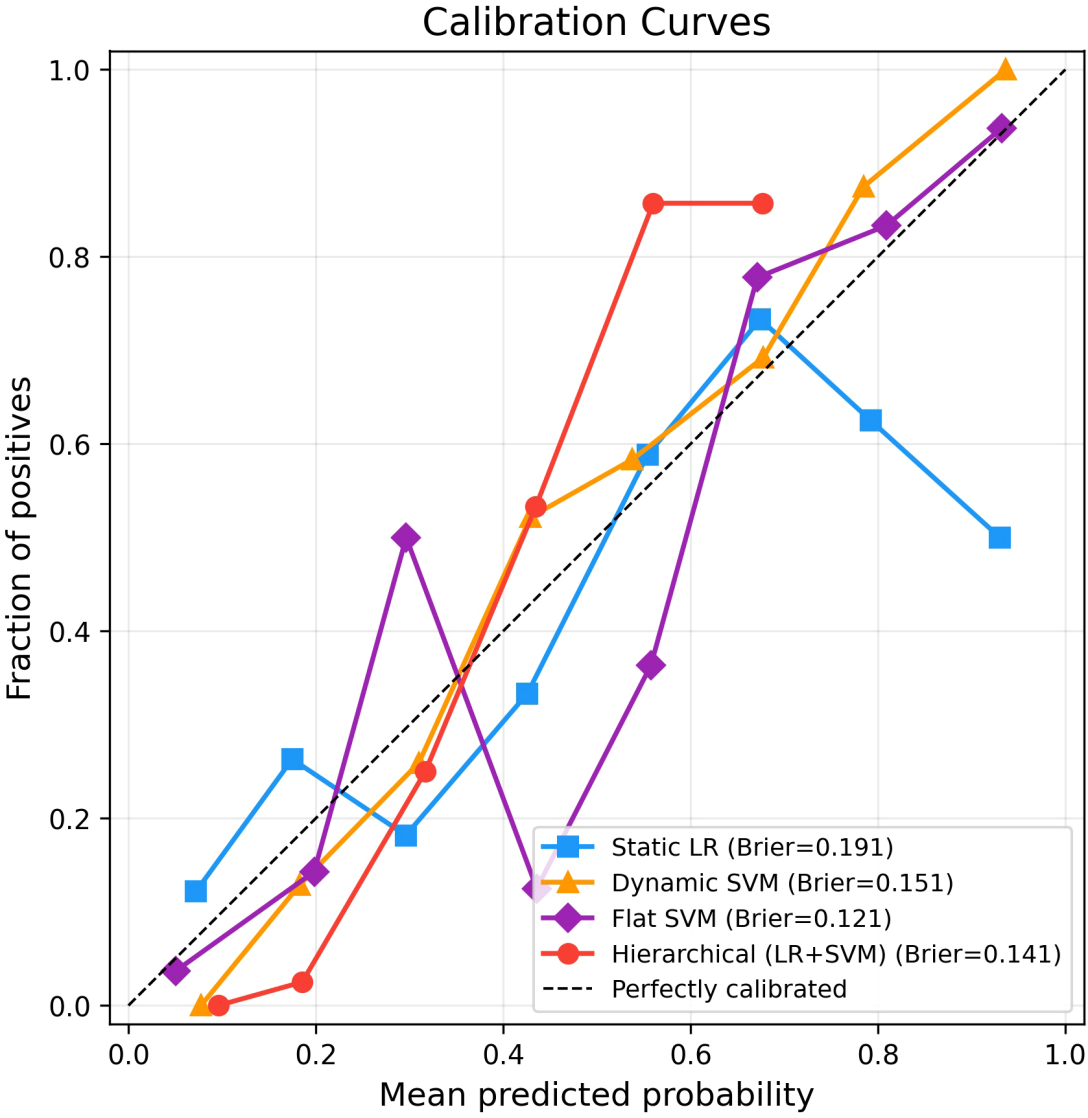
Calibration curves of key models on the independent test set. The dashed diagonal line represents perfect calibration. Brier scores are shown in the legend.

To complement the discrimination metrics reported above, we examined the calibration of the four key models. **Table 10** summarises the Brier score, expected calibration error (ECE), calibration slope and calibration intercept for each model, and **Figure 2** shows the corresponding calibration curves.

**Table 10:** Calibration metrics of key models on the independent test set.

Model	Brier score (95% CI)	ECE (95% CI)	Calibration slope	Calibration intercept
Static LR	0.1907 (0.1530–0.2310)	0.0989 (0.0760–0.1829)	0.6482	-0.1453
Dynamic SVM	0.1505 (0.1253–0.1782)	0.0819 (0.0752–0.1575)	1.4936	0.1707
Flat SVM	0.1207 (0.0900–0.1531)	0.0684 (0.0589–0.1421)	1.0294	-0.1477
Hierarchical (LR+SVM)	0.1409 (0.1197–0.1632)	0.1178 (0.0960–0.1826)	2.6942	0.7824

Brier score ranges from 0 (perfect) to 1 (worst); ECE, Expected Calibration Error; ideal calibration slope, 1 and intercept, 0.

The flat SVM exhibited the best calibration overall, with a slope close to unity (1.03), the lowest Brier score (0.121) and the lowest ECE (0.068), indicating that its predicted probabilities closely matched observed event rates across the full range. The dynamic SVM showed acceptable calibration (slope = 1.49, Brier = 0.151), with a tendency to underestimate risk at higher predicted probabilities. The static LR model displayed moderate miscalibration (slope = 0.65), reflecting overly dispersed probability estimates relative to the observed event rates, consistent with the limited discriminative information available from baseline features alone.

The hierarchical LR+SVM model achieved a Brier score of 0.141 but showed the largest calibration deviation (slope = 2.69, ECE = 0.118). Inspection of the calibration curve revealed that the model systematically underestimated risk in the upper probability range: patients with predicted probabilities of 0.50–0.70 had observed event rates exceeding 0.85. This pattern is attributable to the linear probability fusion formula (*R*^∗^ = *αR*_0_ + (1 − *α*)*R*_dyn_), which compresses the output range and prevents predicted probabilities from reaching extreme values. Importantly, this miscalibration affects the absolute accuracy of predicted probabilities but does not diminish the model’s ability to rank patients by risk, as reflected in its superior AUC (0.9048) and sensitivity (0.8542).

## 4 Discussion

This study examined whether a hierarchical machine-learning framework that links admission-time clinical history with repeated nursing observations can predict short-term self-harm and suicidal behaviour in inpatients with schizophrenia. The final model, which combined a regularised logistic regression on static features with an SVM on dynamic features, achieved excellent discrimination on the independent test set (AUC = 0.9048) together with high sensitivity and NPV. In practice, this means that the model can reliably separate patients who do and do not engage in self-harm/suicidal acts during the current admission. When considered alongside our earlier work on violent behaviour using the same data infrastructure (20), these findings indicate that a single hierarchical architecture can be adapted to different adverse outcomes and recover distinct patterns of risk for self-directed versus other-directed harm.

Regarding static admission characteristics, four variables—previous self-harm or suicide attempts, younger age, hopelessness/depression, and higher educational attainment—remained independently associated with in-hospital self-harm/suicidal acts. The dominant role of prior self-harm accords with a substantial literature showing that past attempts are among the strongest predictors of subsequent suicidal behaviour in schizophrenia and other severe mental disorders (1, 2, 6). The inverse association with age is also consistent with meta-analytic evidence that suicide risk is greatest in younger patients and earlier in the course of illness (1, 4). Baseline hopelessness and depressive mood have long been recognised as key drivers of suicidality in psychotic disorders, and our results reinforce this: patients rated as hopeless or depressed at admission had roughly threefold higher odds of in-hospital self-harm/suicidal behaviour (5, 21, 37). The association with higher education may initially appear paradoxical, but fits with previous work suggesting that better education and higher premorbid functioning can be linked to greater insight and higher expectations, which may amplify feelings of loss and hopelessness when severe illness emerges (2, 38). Together, these findings outline a plausible baseline risk profile: younger patients with a history of self-harm and prominent hopeless or depressive mood, and relatively higher educational attainment, warrant particular attention during admission.

Dynamic indicators derived from weekly nursing observations provided additional, proximal warning signals. Six items—negative self-evaluation, self-reported depression, insomnia, crying, talking to self, and reduced conversation with others—remained independently predictive after adjustment. These behaviours align closely with well-established psychological constructs such as self-stigma, perceived worthlessness, subjective depressive distress, sleep disturbance and social withdrawal, all of which have been implicated in the onset and persistence of suicidal ideation and behaviour in schizophrenia (1, 37). The link between insomnia and self-harm/suicidal acts is particularly notable given longitudinal and meta-analytic evidence that sleep problems represent robust, modifiable risk factors for suicidal thoughts and behaviours (39, 40). Detecting this risk via a simple weekly nurse rating highlights the potential value of systematically monitoring sleep in routine ward practice. Likewise, crying and talking to oneself may capture heightened affective dysregulation and internal preoccupation, whereas diminished conversation with others reflects interpersonal disengagement; both patterns are consistent with interpersonal and cognitive models of suicide emphasising thwarted belongingness, perceived burdensomeness and entrapment. Importantly, these dynamic signs are directly observable in everyday clinical care and may be amenable to targeted interventions, such as sleep management, enhanced emotional support and structured social or occupational activities. Our emphasis on nurse-rated behavioural indicators is further supported by a recent cross-sectional study of 131 schizophrenia inpatients, in which the Nurses’ Observation Scale for Inpatient Evaluation emerged as an important predictor of suicide risk in machine-learning models (19). Together, these converging findings suggest that structured nursing observations carry clinically meaningful information for identifying self-harm and suicidal risk in this population.

The hierarchical integration of static and dynamic information yielded clear performance gains relative to models relying on either source alone, and maintained comparable overall discrimination (AUC) to flat models that concatenated all features while achieving substantially higher sensitivity. The optimal fusion weight placed greater emphasis on the dynamic component while still benefiting from the static baseline, suggesting that long-term vulnerability and short-term behavioural fluctuations contribute complementary information and both are needed for accurate short-term prediction. This pattern accords with recent reviews and meta-analyses indicating that models which combine rich clinical histories with longitudinal or behavioural data outperform simpler actuarial tools and unaided clinician judgement in suicide risk prediction (12, 13, 41). Our findings extend these broader trends by showing that, even within a single psychiatric ward, static EMR data and structured weekly nursing observations can be combined into an accurate and interpretable risk model. This advantage of dynamic updating has also been demonstrated in larger psychiatric inpatient cohorts. Kyron et al. (42) used daily self-reported mental health data from over 17,500 psychiatric inpatients and found that models relying solely on static admission information degraded over time, whereas incorporating dynamically updated patient data maintained predictive stability and reduced false-positive rates. Similarly, Sheu et al. (43) developed continuous-time dynamic risk prediction models based on electronic health records of more than 1.7 million patients and showed that modelling risk as a time-varying trajectory rather than a fixed score substantially improved discrimination. Although our weekly nursing observations are less frequent than daily self-reports or continuous EHR updates, the principle is the same: separating long-term vulnerability from short-term state changes and recombining them yields a more faithful representation of how risk evolves during inpatient care. From a complementary perspective, Simon et al. (44) demonstrated that even structured EHR data alone can achieve meaningful risk stratification (AUC = 0.823) for suicide death following emergency department visits; our results suggest that adding dynamic ward-based behavioural observations to such baseline information can push discriminative performance further. Importantly, a direct comparison with flat models that concatenated all 57 features into a single input vector showed that the hierarchical model did not sacrifice overall discriminative ability: the best flat model (SVM, AUC = 0.9022) performed comparably to the hierarchical LR_static_+SVM_dynamic_ model (AUC = 0.9048). However, the hierarchical model achieved substantially higher sensitivity (0.8542 vs. 0.6667) at the default decision threshold, a critical advantage in self-harm and suicide screening where undetected high-risk patients may face life-threatening consequences. This performance pattern is consistent with the hypothesis that explicitly separating static vulnerability from dynamic behavioural change allows the fusion step to calibrate the decision boundary in a way that prioritises the detection of at-risk individuals. Notably, the hierarchical model achieved this favourable sensitivity–specificity balance at the default 0.5 threshold without any threshold optimisation, suggesting that its structural advantage is intrinsic rather than an artefact of threshold selection. In clinical deployment, the operating threshold should be adapted to the intended use case: a lower threshold would further increase sensitivity for broad ward-level screening, whereas a higher threshold might be appropriate when the goal is to allocate intensive resources to the highest-risk patients. Such threshold calibration, together with cost-effectiveness analysis, should be addressed in prospective implementation studies. Beyond prediction metrics, the hierarchical architecture offers a structural advantage in clinical interpretability: the separate static and dynamic probability estimates (*R*_0_ and *R*_dyn_) allow clinicians to trace whether a given patient’s risk is driven primarily by historical factors or by recent symptomatic deterioration, supporting more targeted and transparent risk communication and intervention planning.

Comparison with our previous hierarchical model for violent behaviour further illustrates how the same analytical framework can reveal different risk pathways. In the violence model, static risk was dominated by prior violence, manic symptoms and high-risk command hallucinations, whereas dynamic predictors included anger expression, rule-breaking and poor cooperation with staff (20). These features typify externalising aggression, disinhibition and hostility. By contrast, the present self-harm/suicide model is characterised by internalising and self-directed phenomena: prior self-harm, hopelessness/depression, negative self-evaluation, crying, talking to self, insomnia and social withdrawal. This divergence supports the view that violent and self-harming behaviours in schizophrenia, although sharing some nonspecific risk factors (e.g. younger age or substance use), are largely driven by different psychopathological mechanisms—externalising versus internalising pathways—consistent with comparative work on violence and suicidality in psychosis (45–48). That the hierarchical framework naturally recovers these distinct patterns when applied to different endpoints adds construct validity to both models.

Several implications for clinical practice follow. Admission information can be used to stratify patients into baseline risk strata and to identify those for whom early, enhanced monitoring or preventive measures may be justified, particularly patients with a history of self-harm and marked hopelessness or depression. The dynamic nursing observations provide a practical mechanism for ongoing risk surveillance: rising negative self-evaluation, self-reported depression, crying, insomnia or self-talk, together with progressive social withdrawal, should prompt review of safety plans, therapeutic engagement and environmental supports. The high NPV of the hierarchical model indicates that patients classified as low risk are unlikely to self-harm during their stay, which may help clinicians prioritise resources without compromising safety. More broadly, our results add to accumulating evidence that machine-learning tools can complement rather than replace clinical judgement by offering consistent, data-driven risk estimates and supporting more structured and transparent risk formulation (49).

This work also has limitations. The study was retrospective and conducted at a single psychiatric hospital in Liaoning Province; generalisability to other settings with different patient populations, documentation practices or treatment structures is therefore uncertain. In particular, differences in nursing observation protocols, staffing ratios, ward environments and patient demographics across institutions may affect both the prevalence of self-harm/suicidal behaviour and the discriminative value of individual predictors. Cultural and regional factors—such as attitudes towards mental illness, thresholds for hospitalisation and documentation conventions—could further influence the transferability of our findings. Independent external validation in multicentre cohorts with diverse clinical settings will be essential before routine implementation is considered (50). This concern is not unique to our study. A recent systematic review of 167 self-harm and suicide prediction models found that only 8% had undergone external validation, and that discrimination typically declined when models were tested in new populations (51). Moreover, a meta-analysis by Spittal et al. (18) concluded that the overall evidence quality for machine-learning suicide prediction remains mixed, with many studies exhibiting high or unclear risk of bias and predictive accuracy that does not consistently surpass traditional risk scales. These observations underscore the need for cautious interpretation of our results: although the hierarchical model shows promising internal performance, it should be viewed as providing preliminary evidence for a specific inpatient setting rather than a ready-to-deploy clinical tool. Prospective validation would also allow assessment of model calibration over time and under different treatment regimens, which cannot be addressed in a retrospective design. Our calibration analysis showed that, while the flat SVM produced well-calibrated probability estimates (slope = 1.03), the hierarchical model exhibited notable miscalibration (slope = 2.69), with predicted probabilities systematically underestimating actual event rates in the upper range. This is an inherent property of linear probability averaging, which compresses the output range. As a result, the hierarchical model’s predicted probabilities should not be interpreted at face value as absolute risk estimates; rather, the model is most appropriately used as a ranking and screening tool. If absolute probability estimates are required for clinical decision-making, *post-hoc* recalibration methods such as isotonic regression or Platt scaling could be applied, ideally on independent external data. Until such validation is available, the model remains at the internal validation stage and should not be considered ready for routine clinical deployment. In its current form, it is best regarded as a proof-of-concept decision-support tool that complements, rather than replaces, clinical judgement and established risk assessment procedures. Although our sample and event numbers compare favourably with many previous studies on suicidality in schizophrenia, they remain modest for complex machine-learning models, so some overfitting—particularly for the more flexible algorithms—cannot be excluded. The outcome definition combined non-fatal self-harm and suicide attempts irrespective of method or inferred intent. Although this inclusive approach is consistent with WHO and NICE frameworks (27, 28) and is pragmatically justified by the difficulty of reliably ascertaining suicidal intent from retrospective charts (1, 6), it has implications for interpretation. The risk factors identified reflect an average effect across both forms of self-directed harm, and predictors specific to one subtype may have been diluted; moreover, a high-risk classification should be understood as indicating elevated risk of self-harm broadly defined, not exclusively suicidal behaviour. Future studies using prospective designs with structured intent assessments could examine whether separate models for suicidal versus non-suicidal self-injury yield distinct and more actionable risk profiles. In addition, patients whose first self-harm or suicidal act occurred during the initial week of hospitalisation were excluded because they lacked sufficient pre-event nursing observation data for the dynamic model. This exclusion may introduce selection bias, as these patients could represent an acutely high-risk subgroup whose risk profile differs from that of patients who self-harm later in the admission. Consequently, the model’s performance estimates may not generalise to this early-onset group. However, this constraint is inherent to any predictive framework that relies on longitudinal behavioural input collected after admission. In practice, the hierarchical architecture offers a natural mitigation: for newly admitted patients who lack dynamic observations, the static sub-model (*R*_0_) can provide an initial risk estimate based on clinical history alone, with the full hierarchical score activated once sufficient nursing data have accumulated. Dynamic observations were collected only once per week and were averaged into a single summary vector per patient, which may miss both within-week fluctuations and longer-term temporal trends; however, the relatively short and homogeneous observation window in our cohort (4.46 ± 1.87 weeks) suggests that simple averaging retains the main severity signal. More frequent sampling, trend-based features or sequential modelling architectures could capture impending crises more precisely in future work (52, 53). Finally, as with most predictive modelling studies, our analyses are correlational and should not be interpreted as establishing causal effects; causal inference–oriented approaches may be useful in future work when considering these variables as potential intervention targets (54).

Looking forward, several extensions are worth exploring. Methodologically, multi-task models could be developed to predict both self-harm/suicidal behaviour and interpersonal violence within a single framework, enabling explicit investigation of shared and outcome-specific risk pathways. Incorporating additional data modalities—such as free-text clinical notes processed with natural language processing, neurocognitive measures, neuroimaging or biological markers—may further enhance performance and help clarify underlying mechanisms (13, 15). Clinically, prospective implementation studies are needed to evaluate how hierarchical risk scores influence clinician behaviour, patient outcomes and ward workflows, and to identify best practices and safeguards for integrating such tools into routine care.

In summary, our findings provide preliminary single-centre evidence that a hierarchical machine-learning model combining static clinical history with dynamic behavioural observations can accurately predict short-term self-harm and suicidal behaviour in hospitalised patients with schizophrenia, with the additional advantage of providing clinically interpretable, decomposed risk estimates that distinguish enduring vulnerability from acute behavioural warning signs. Together with our previous work on violence prediction, this suggests that data-driven hierarchical risk assessment frameworks can be tailored to different adverse outcomes and provide nuanced, clinically interpretable insights into both self-directed and other-directed risk. 

## Data Availability

The datasets analyzed in this study are not publicly available because they contain sensitive clinical information and are subject to institutional data governance restrictions. De-identified data may be made available by the corresponding authors upon reasonable request and subject to approval by the Ethics Committee of Liaoning Provincial Mental Health Center and the host institution. The analysis code is available from the corresponding authors upon reasonable request.
